# An Equity Analysis of Zero-Dose Children in India Using the National Family Health Survey Data: Status, Challenges, and Next Steps

**DOI:** 10.7759/cureus.35404

**Published:** 2023-02-24

**Authors:** Gunjan Taneja, Eshita Datta, Mahima Sapru, Mira Johri, Kapil Singh, Harkabir S Jandu, Shyamashree Das, Arindam Ray, Kayla Laserson, Veena Dhawan

**Affiliations:** 1 Infectious Disease Cluster, India Country Office, Bill & Melinda Gates Foundation, New Delhi, IND; 2 Social Impact Practice, Evalueserve, New Delhi, IND; 3 School of Public Health, University of Montreal, Montreal, CAN; 4 Immunization Division, Ministry of Health and Family Welfare, Government of India, New Delhi, IND; 5 Strategy, Clinton Health Access Initiative, New Delhi, IND

**Keywords:** zero dose, vaccination, national family health survey, immunization, equity

## Abstract

Background

While immunization programs across the world have made considerable progress, children and communities continue to be beyond the reach of healthcare services. Globally, they are now referred to as zero-dose (ZD) children (those who have not received a single dose of diphtheria, pertussis, and tetanus-containing vaccine). Pre-COVID-19 pandemic analyses suggest that nearly 50% of vaccine-preventable deaths occur among ZD children. Two-thirds of these children live in extremely poor households suffering from multiple deprivations including lack of access to reproductive health services, water, and sanitation. Hence, ZD children have now been prioritized as a key cohort for identification and integration with the health systems as we build back from the pandemic.

Methodology

Extracting data from the last two National Family Health Survey (NFHS) rounds (NFHS 4, 2015-2016 and NFHS 5, 2019-2021), this study aims to ascertain the status of ZD children aged 12-23 months in India, the challenges, and the necessary action agenda going forward. Data were analyzed for equity determinants such as gender, place of residence, religion, birth order, caste, and mother’s schooling. Key determinants included the change in ZD prevalence at the national, state, and district levels; variations across equity parameters and states with maximum improvements; and disparity across these indicators. A correlation analysis was also conducted to understand the nature of the association between ZD prevalence and critical maternal and child health indicators.

Results

The overall ZD prevalence between the two rounds was reduced by 4.1% (10.5-6.4%). A total of 26 states in the country reported a ZD prevalence of <10% in NFHS 5 compared to 18 in NFHS 4. In total, 324 districts reported a ZD prevalence of <5%, and 145 districts reported a prevalence of >10%. The equity parameters reflected a slow-footed reduction among ZD for girl children, across urban geographies, firstborn children, mothers with 12 or more years of schooling, and children in families with the highest wealth quintiles. A negative correlation accentuated between the two NFHS rounds was established between first-trimester registration, four or more antenatal visits, institutional deliveries, and ZD prevalence.

Conclusions

The findings point toward sustained improvement across key equity parameters, however, challenges do exist. Moreover, the impact of the pandemic on immunization programs across the globe and in India is bound to halt and reverse the progress and potentiate further inequities. It is thus imperative that continued and augmented efforts are continued to identify, integrate, and immunize ZD children, families, and communities.

## Introduction

While immunization programs across the world have made considerable progress, children and communities continue to be beyond the reach of healthcare services. Globally, they are now referred to as zero-dose (ZD) children (those who have not received a single dose of diphtheria, pertussis, and tetanus-containing vaccine) [1]. ZD children and communities are the most marginalized and deprived, with pre-pandemic analyses suggesting that nearly 50% of vaccine-preventable deaths occur among them [2]. Two-thirds of these children live in extremely poor households suffering from multiple deprivations including lack of access to reproductive health services, water, and sanitation [3].

Hence, recent efforts recognize the need for reaching out to ZD children. The Immunization Agenda 2030 aims to half the number of ZD children by 2030 [4], and the new GAVI 5.0 strategy envisages health systems to sustainably reach all ZD and under-immunized children and their communities with the full range of vaccines as the first step toward providing integrated primary healthcare (PHC) services [5]. Moreover, the second progress report of the Sustainable Development Goal (SDG) 3 Global Action Plan recognizes ZD children as a core part of its approach toward equitable and resilient recovery from the pandemic [6].

In line with the above efforts, the Government of India has prioritized efforts to reach out to ZD children, with the recent GAVI support to the country identifying 143 districts across 11 states for focused support [7]. In addition, while India has shown considerable progress over the years [8], it still accounted for 1.6 million ZD children in 2019 [7]. Thus, it is imperative that an in-depth analysis of ZD children in India is undertaken to identify critical determining factors and the interventions necessary to address existing challenges.

## Materials and methods

This article presents the findings of secondary analyses of data sourced from the National Family Health Survey (NFHS). NFHS is a large-scale, multi-round survey conducted in a representative sample of households throughout India [9]. It is used as a reference to assess the progress the country has achieved across a multitude of programs. These include family planning, maternal and delivery care, child vaccinations, treatment of childhood diseases, feeding practices and nutrition status of children, nutrition status of adults, anemia among children and adults, blood sugar and hypertension level among adults, tobacco and alcohol consumption, screening for cancer among adults, knowledge on HIV/AIDs among adults, women empowerment, and gender-based violence. The data are publicly available in the form of factsheets, state reports, and raw data for national, state, and district levels.

Data for immunization are available for the point of service (public and private) and the coverage estimates for individual antigens (BCG), hepatitis B birth dose, pentavalent (DPT, hepatitis B, and *Haemophilus influenzae* type b), oral polio vaccine, measles-containing vaccine (MCV), and rotavirus vaccine (RVV). In addition, data are available for key equity parameters including gender, place of residence, religion, birth order, caste, and mother’s schooling. While the NFHS factsheets provide data on key coverage indicators, the equity indicators are included as part of the state reports. The state reports also provide data on other program indicators, the impact of which can be assessed on the immunization program. Coverage of key immunization indicators for districts is available in the state reports.

Using equity differentiated data from NFHS state reports, this article aims to analyze the progress achieved across states of the country in reaching out to ZD children between the last two NFHS rounds (NFHS 5, 2019-2021 and NFHS 4, 2015-2016). ZD proportions were measured using pentavalent 1 coverage as the indicator. The key determinants studied include the change in ZD prevalence at the national, state, and district levels; the proportion of change in equity determinants; the states with maximum improvements; the maximum disparity across these indicators; and the overall reduction in disparities. The data were interpreted in the form of tables and maps. The maps were created using choropleth maps on Datawrapper [10] and the map feature on Microsoft Excel.

A correlation analysis was conducted to understand the nature of the association between ZD prevalence and critical maternal and child health (MCH) indicators which include four or more antenatal care (ANC) visits, the timing of pregnancy registration, institutional delivery (birth at a health facility), children under five years old who are stunted (height-for-age), and children under five years old who are wasted (weight-for-height). For each of these indicators, data were collated for both the NFHS rounds (NFHS 5 and NFHS 4), and the Pearson correlation coefficient was obtained using the following formula: 

r=∑[(xi-x ¯)(yi-ȳ)] ⁄ √[∑(xi-x ¯)^2 ∑(yi-ȳ)^2]

with the coefficient value r signifying the strength and direction of correlation between the two variables. The strength of association as per the correlation coefficient values was interpreted as follows: no association: 0, weak association: (±) 0.1 to less than 0.3, moderate association: (±) 0.3 to less than 0.5, strong association: (±) 0.5 to less than 1, and perfect association: (±) 1.

## Results

The overall ZD prevalence between the two NFHS rounds has come down by 4.1% (10.5-6.4%), with an annual cohort of 26 million, which translates to 1.7 million ZD children in the country. Five states in the country, namely, Bihar, Madhya Pradesh, Maharashtra, Rajasthan, and Uttar Pradesh, which account for almost 55% of the birth cohort, account for about 63% of the ZD children.

**Figure 1:**
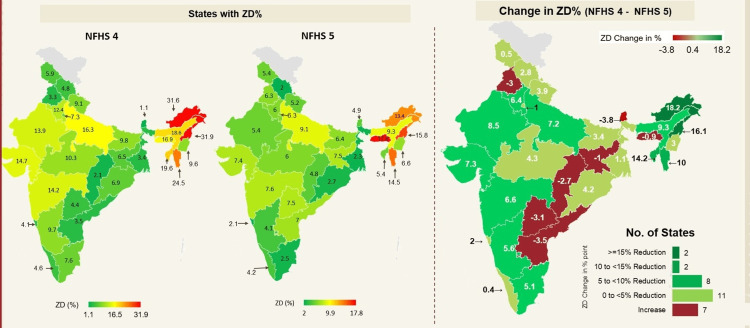
Prevalence and change in ZD children across states of India (NFHS 4 to NFHS 5). This analysis is based on data from 30 states of the country. ZD = zero dose; NFHS = National Family Health Survey

Data are available and comparable for 30 states in the country between the two rounds (NFHS 5 and NFHS 4). In NFHS 5, nine states reported a ZD prevalence of 0 to <5%, 17 states a prevalence of 5% to <10%, and two states each reported a prevalence of 10% to <15% and 15% and above. The corresponding figures for NFHS 4 were nine, nine, five, and seven, respectively. ZD prevalence in the country ranges from 17.8% in Meghalaya to 2% in Himachal Pradesh, as per NFHS 5, and this ranged from 31.9% (Nagaland) to 1.1% (Sikkim) as per NFHS 4. While three states (Arunachal Pradesh, Nagaland and Tripura) have shown the maximum reduction between the two NFHS rounds, Andhra Pradesh, Chhattisgarh, Jharkhand, Meghalaya, Sikkim, Punjab, and Telangana have reported an increase in ZD prevalence between NFHS 4 and NFHS 5. In addition, despite substantial reduction, northeastern states of the country continue to have the highest ZD prevalence across the country, and this reduction is attributable to the high baselines across these states in NFHS 4 (Figure 1).

**Figure 2:**
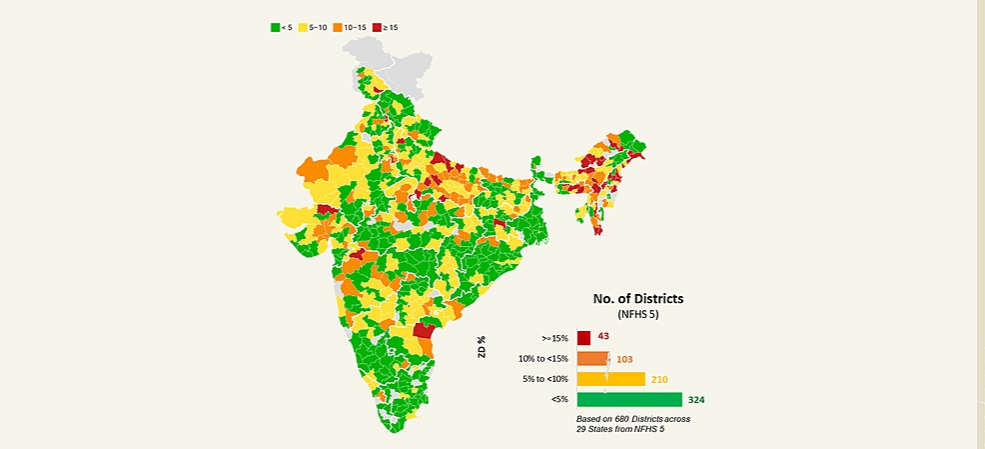
District-wise prevalence of ZD children in India (NFHS 5). ZD = zero dose; NFHS = National Family Health Survey

Data were analyzed for 680 districts for which data is available in NFHS 5, out of which 324 districts reported a prevalence of 0 to <5%, 210 a prevalence of 5% to <10%, 103 a prevalence of 10% to <15%, and 42 districts a prevalence of 15% and higher. Nine of these 42 districts are in the state of Arunachal Pradesh, eight in Uttar Pradesh, seven in Nagaland, four in Assam, three each in Meghalaya and Mizoram, and one district each across the states of Andhra Pradesh, Gujarat, Haryana, Jammu & Kashmir, Jharkhand, Maharashtra, Manipur, Punjab, and Telangana (Figure 2).

Progress on equity determinants: national level

ZD prevalence at the national level has been analyzed for key equity determinants. The ZD prevalence for the male child has reduced from 10.3% to 6.0% and for female child from 10.7% to 6.8%, reflecting a decline of 4.3% and 3.9%, respectively. Regarding the place of residence, the prevalence has come down by 2.2% in urban areas (9.7% to 7.5%) and 4.9% (10.8% to 5.9%) in rural areas of the country. The reduction among Muslim children is 6.7% (16.0% to 9.3%) between the two rounds compared to 3.5% (9.3% to 5.8%) for Hindu children. Among children belonging to Scheduled Caste (SC) the prevalence has reduced from 9.8% to 6.1% (3.7%) between the two rounds, similarly the reduction has been from 13.6% to 7.1% (6.5%) for Scheduled Tribes (ST), 10.1% to 6.1% (4.0%) for Other Backward Class (OBC), and 10.3% to 6.7% (3.6%) among others. There has been a 2.4% reduction in ZD children among the first-born child (8.1% to 5.7%), 6.3% (15.9% to 9.6%) in birth orders four to five, and 9.4% (26.0% to 16.6%) in birth orders 6 and higher. Among mothers with no schooling, the ZD prevalence has reduced by 7.0% (17.1% to 10.1%) and mothers with 12 or more years of schooling by 1.2% (6.0% to 4.8%). The ZD prevalence has reduced from 7.1% to 5.5% among the highest wealth quintile, from 7.0% to 5.1% in the fourth highest quintile, from 8.8% to 5.4% in the middle quintile, 11.4% to 6.7% in the second lowest, and from 15.8% to 8.4% in the lowest wealth quintile. This translates to a reduction of 1.6%, 1.9%, 3.4%, 4.7%, and 7.4%, respectively, among the five groups (Table 1).

**Table 1:** ZD prevalence and change across equity parameters: national level (NFHS 4 to NFHS 5). ZD = zero dose; NFHS = National Family Health Survey

Equity parameter	NFHS 4 (%)	NFHS 5 (%)	Difference (%)
Gender			
Female	10.7	6.8	3.9
Male	10.3	5.6	4.3
Mother’s schooling			
12 or more years	6.0	4.8	1.2
No schooling	17.1	10.1	7.0
Birth order			
1	8.1	5.7	2.4
4–5	15.9	9.6	6.3
6 or more	26.0	16.6	9.4
Religion			
Hindu	9.3	5.8	3.5
Muslim	16.0	9.3	6.7
Caste			
Scheduled Caste	9.8	6.1	3.7
Scheduled Tribe	13.6	7.1	6.5
Other Backward Class	10.1	6.1	4.0
Others	10.3	6.7	3.6
Residence			
Urban	9.7	7.5	2.2
Rural	10.8	5.9	4.9
Wealth			
Highest quintile	7.1	5.5	1.6
Fourth quintile	7.0	5.1	1.9
Middle quintile	8.8	5.4	3.4
Second quintile	11.4	6.7	4.7
Lowest quintile	15.8	8.4	7.4

Progress on equity determinants: state level

The article analyses the progress on key equity determinants across states of the country (Table 2).

**Table 2:** State-wise ZD prevalence and change across equity parameters (NFHS 4 to NFHS 5). ZD = zero dose; NFHS = National Family Health Survey

State	DTP 1 / Penta 1 (%)																																																																																																		
	Total					Gender										Birth order																	Residence										Mother's Schooling																	Religion																		Caste																					
	NFHS 5		NFHS 4		Difference	NFHS 5				NFHS 4				Difference		NFHS 5							NFHS 4							Difference			NFHS 5				NFHS 4				Difference		NFHS 5								NFHS 4						Difference			NFHS 5								NFHS 4								Difference		NFHS 5									NFHS 4									Difference			
	DTP 1 / Penta 1 (%)	ZD (%)	DTP 1 / Penta 1 (%)	ZD (%)	Reduction ZD% (NFHS 4-5)	Male DTP 1 / Penta 1 (%	Male (ZD %)	Female DTP 1 / Penta 1 (%	Female (ZD%)	Male DTP 1 / Penta 1 (%	Male (ZD %)	Female DTP 1 / Penta 1 (%	Female (ZD%)	Male (NFHS 4 - 5)	Female (NFHS 4-5)	1	1 ZD (%)	2,3	4,5	4,5 ZD (%)	6 and more	6 and more ZD (%)	1	1 ZD (%)	2,3	4,5	4,5 ZD (%)	6 and more	6 and more ZD (%)	1 (NFHS 4-5)	4,5 (NFHS 4-5)	6 and more (NFHS 4-5)	Urban	Urban (ZD%)	Rural	Rural (ZD%)	Urban	Urban (ZD%)	Rural	Rural (ZD%)	Urban (NFHS 4-5)	Rural (NFHS 4-5)	No schooling	No Schooling (ZD%)	<5 years	5-7 years	8-9 years	10- 11 years	12 or more years	12 or more years (ZD%)	No schooling	No Schooling (ZD%)	<5 years	5-9 years	10- 11 years	12 or more years	12 or more years (ZD%)	No Schooling (NFHS 4-5)	12 or more years (NFHS 4-5)	Hindu	Hindu ZD (%)	Muslim	Muslim ZD (%)	Christian	Buddhist/Neo Buddhist	Others	Sikh	Hindu	Hindu ZD (%)	Muslim	Muslim ZD (%)	Christian	Buddhist/Neo Buddhist	Others	Sikh	Hindu (NFHS 4-5)	Muslim (NFHS 4-5)	SC	SC ZD (%)	ST	ST ZD (%)	OBC	OBC ZD (%)	Others	Others ZD (%)	Don't Know	SC	SC ZD (%)	ST	ST ZD (%)	OBC	OBC ZD (%)	Others	Others ZD (%)	Don't Know	SC (NFHS 4-5)	ST (NFHS 4-5)	OBC (NFHS 4-5)	Others (NFHS 4-5)
Andhra Pradesh	93	7	96.5	3.5	-3.5	93.1	6.9	92.9	7.1	96.2	3.8	96.9	3.1	-3.1	-4	93.8	6.2	93.2	NA		NA		97.5	2.5	96	94.2	5.8	NA		-3.7			89.4	10.6	94.6	5.4	97.7	2.3	96.1	3.9	-8.3	-1.5	92	8	NA	90.9	93.6	94.4	94	6	94.1	5.9	90.5	97	96.5	99.1	0.9	-2.1	-5.1	92.4	7.6	95.7	4.3	95.1	NA	NA	NA	97.6	2.4	95.1	4.9	88.9	NA	NA	NA	-5.2	0.6	92.9	7.1	92.9	7.1	92.3	7.7	95.2	4.8	NA	94.5	5.5	92.5	7.5	98.3	1.7	95.2	4.8	NA	-1.6	0.4	-6	0
Arunachal Pradesh	86.6	13.4	68.4	31.6	18.2	86.8	13.2	86.4	13.6	69.4	30.6	67.2	32.8	17.4	19.2	87.1	12.9	87	85.2	14.8	76	24	79.4	20.6	70.2	55.6	44.4	40.4	59.6	7.7	29.6	35.6	91.9	8.1	85.8	14.2	76.4	23.6	65.9	34.1	15.5	19.9	79	21	83	89.6	87.4	89.6	90.5	9.5	51.3	48.7	61.4	74.6	83	86.8	13.2	27.7	3.7	88.9	11.1	NA		85.9	80.3	91.7	NA	79.2	20.8	NA		58.1	75.9	75.2	NA	9.7		82.9	17.1	85.8	14.2	92.3	7.7	88.5	11.5	NA	78.2	21.8	61.4	38.6	83.2	16.8	88.7	11.3	NA	4.7	24.4	9.1	-0.2
Assam	90.7	9.3	81.4	18.6	9.3	90.4	9.6	91.1	8.9	83.2	16.8	79.5	20.5	7.2	11.6	91.4	8.6	91.7	84.9	15.1	71.7	28.3	85.6	14.4	80.8	71.7	28.3	65.1	34.9	5.8	13.2	6.6	91.4	8.6	90.6	9.4	94.3	5.7	79.9	20.1	-2.9	10.7	86.5	13.5	94	89.6	91.3	92.7	91.6	8.4	71.3	28.7	82.4	83.8	85.6	86.4	13.6	15.2	5.2	92.7	7.3	88.6	11.4	86.2	NA	NA	NA	87.8	12.2	72.8	27.2	94.9	NA	NA	NA	4.9	15.8	90.1	9.9	92.6	7.4	94.7	5.3	89	11	NA	89.7	10.3	86.9	13.1	87	13	76.2	23.8	NA	0.4	5.7	7.7	12.8
Bihar	93.6	6.4	90.2	9.8	3.4	95.2	4.8	91.8	8.2	89.5	10.5	90.9	9.1	5.7	0.9	93.2	6.8	94.5	94.2	5.8	83.3	16.7	91.4	8.6	91	88	12	84.6	15.4	1.8	6.2	-1.3	93	7	93.6	6.4	91.8	8.2	90	10	1.2	3.6	92.5	7.5	93.8	94.7	93.9	95.2	94.1	5.9	87.4	12.6	92.1	94	89.6	96	4	5.1	-1.9	94.6	5.4	88.4	11.6	NA	NA	NA	NA	91.6	8.4	84	16	NA	NA	NA	NA	3	4.4	93.4	6.6	94.5	5.5	93.9	6.1	92.7	7.3	89.1	89.1	10.9	89.5	10.5	91	9	88.6	11.4	NA	4.3	5	2.9	4.1
Chhattisgarh	95.2	4.8	97.9	2.1	-2.7	95.3	4.7	95.1	4.9	98.5	1.5	97.4	2.6	-3.2	-2.3	96.4	3.6	94.5	94.1	5.9	89.4	10.6	99.5	0.5	97.1	98.4	1.6	93.1	6.9	-3.1	-4.3	-3.7	91.8	8.2	96.1	3.9	98.5	1.5	97.8	2.2	-6.7	-1.7	94	6	97.3	93.9	94.1	96.9	96.3	3.7	96.1	3.9	98.9	98.2	99.3	98.9	1.1	-2.1	-2.6	NA		NA		NA	NA	NA	NA	98.1	1.9	100	0	NA	NA	NA	NA			95.5	4.5	95	5	95.5	4.5	92.9	7.1	NA	99.7	0.3	96.9	3.1	97.9	2.1	99.3	0.7	NA	-4.2	-1.9	-2.4	-6.4
Goa	97.9	2.1	95.9	4.1	2	NA		NA		96.7	3.3	95.2	4.8	3.3		NA		NA	NA		NA		96.8	3.2	94.4	NA		NA					NA		NA		94	6	100	0			NA		NA	NA	NA	NA	NA		NA		NA	NA	NA	NA				NA		NA		NA	NA	NA	NA	NA		NA		NA	NA	NA	NA			NA		NA		NA		NA		NA	NA		NA		NA		NA		NA				
Gujarat	92.6	7.4	85.3	14.7	7.3	93	7	92.2	7.8	85.5	14.5	85.1	14.9	7.5	7.1	93.2	6.8	92.3	91.4	8.6	NA		90.6	9.4	82.2	82.5	17.5	63.6	36.4	2.6	8.9		92.5	7.5	92.7	7.3	87.4	12.6	83.8	16.2	5.1	8.9	90.1	9.9	89.2	93.3	95.5	92.5	93.2	6.8	75.7	24.3	79.6	86.3	94.5	92.1	7.9	14.4	1.1	92.5	7.5	93	7	NA	NA	NA	NA	85.3	14.7	84.4	15.6	NA	NA	NA	NA	7.2	8.6	94.5	5.5	92.5	7.5	92.9	7.1	91.7	8.3	NA	86	14	86.3	13.7	85.2	14.8	86.8	13.2	69.5	8.5	6.2	7.7	4.9
Haryana	94	6	87.6	12.4	6.4	92.8	7.2	95.2	4.8	88.4	11.6	86.7	13.3	4.4	8.5	94.8	5.2	94.3	91	9	81.5	18.5	89.5	10.5	88	80.3	19.7	71.3	28.7	5.3	10.7	10.2	94.5	5.5	93.7	6.3	85.6	14.4	88.8	11.2	8.9	4.9	88.7	11.3	93.4	92.7	95.9	96.4	94.9	5.1	75.1	24.9	78.1	89.8	93.4	93.8	6.2	13.6	1.1	95.2	4.8	83.9	16.1	NA	NA	NA	96.9	90.3	9.7	65.5	34.5	NA	NA	NA	100	4.9	18.4	94.2	5.8	93.9	6.1	NA		94.2	5.8	NA	87.1	12.9	NA		88.6	11.4	86.4	13.6	NA	7.1			7.8
Himachal Pradesh	98	2	95.2	4.8	2.8	99.3	0.7	96.5	3.5	96.3	3.7	94	6	3	2.5	96.9	3.1	98.9	NA		NA		95.9	4.1	94.6	NA		NA		1			97.7	2.3	98	2	90.5	9.5	95.6	4.4	7.2	2.4	NA		NA	100	99.9	96.9	97.5	2.5	NA		NA	96.3	95.8	94.6	5.4		2.9	97.9	2.1	NA		NA	NA	95.4	NA	95.1	4.9	NA		NA	98.3	NA	NA	2.8		98.1	1.9	99.8	0.2	95.9	4.1	98.4	1.6	NA	98.2	1.8	99.7	0.3	96.9	3.1	92.5	7.5	NA	-0.1	0.1	-1	5.9
Jammu & Kashmir	94.6	5.4	94.1	5.9	0.5	93.8	6.2	95.6	4.4	92.8	7.2	95.4	4.6	1	0.2	96.6	3.4	93.4	90.5	9.5	NA		95.4	4.6	94.2	88.9	11.1	88.1	11.9	1.2	1.6		90.5	9.5	95.9	4.1	96.6	3.4	93.2	6.8	-6.1	2.7	96.1	3.9	NA	92.5	95	96.5	93.6	6.4	91.5	8.5	86.9	95.3	92.7	97.4	2.6	4.6	-3.8	94.5	5.5	94.7	5.3	NA	NA	NA	NA	93.1	6.9	94.2	5.8	NA	NA	98.9	NA	1.4	0.5	97.5	2.5	94.8	5.2	95.9	4.1	93.9	6.1	NA	93.1	6.9	91	9	98.6	1.4	94.5	5.5	NA	4.4	3.8	-2.7	-0.6
Jharkhand	92.5	7.5	93.5	6.5	-1	91.8	8.2	93.4	6.6	93.6	6.4	93.5	6.5	-1.8	-0.1	94.5	5.5	91.3	92.6	7.4	84.8	15.2	95	5	94.9	88.6	11.4	74.9	25.1	-0.5	4	9.9	87.2	12.8	93.5	6.5	95.5	4.5	93.1	6.9	-8.3	0.4	91.5	8.5	94.2	91.6	92.7	93.1	93.4	6.6	89	11	90.9	95.7	97.1	98.1	1.9	2.5	-4.7	93.5	6.5	87	13	89.2	NA	94.5	NA	94.4	5.6	91.6	8.4	90.2	NA	91.5	NA	-0.9	-4.6	92.3	7.7	90.4	9.6	93.6	6.4	92.6	7.4	NA	92	8	92.6	7.4	94.7	5.3	92.3	7.7	NA	0.3	-2.2	-1.1	0.3
Karnataka	95.9	4.1	90.3	9.7	5.6	94.6	5.4	97.4	2.6	88	12	92.8	7.2	6.6	4.6	95.6	4.4	96.8	91.2	8.8	NA		91.8	8.2	88.7	90.6	9.4	NA		3.8	0.6		95	5	96.4	3.6	86.9	13.1	92.9	7.1	8.1	3.5	96.1	3.9	100	92.4	98	96.5	95.9	4.1	86.5	13.5	90.2	91.4	90.1	91.5	8.5	9.6	4.4	96.1	3.9	96.3	3.7	NA	NA	NA	NA	90.2	9.8	91.8	8.2	85	NA	NA	NA	5.9	4.5	97.9	2.1	94.2	5.8	96	4	94.4	5.6	NA	90.8	9.2	75.3	24.7	93.9	6.1	89.2	10.8	NA	7.1	18.9	2.1	5.2
Kerala	95.8	4.2	95.4	4.6	0.4	96.6	3.4	94.9	5.1	94.8	5.2	96.1	3.9	1.8	-1.2	95.8	4.2	95.9	NA		NA		97.1	2.9	93.3	NA		NA		-1.3			95.9	4.1	95.7	4.3	96.1	3.9	94.8	5.2	-0.2	0.9	NA		NA	NA	86.1	97.8	96.7	3.3	NA		NA	91.7	91.5	97.4	2.6		-0.7	97.4	2.6	93.2	6.8	98.2	NA	NA	NA	96.9	3.1	93.8	6.2	95	NA	NA	NA	0.5	-0.6	94.3	5.7	NA		95	5	97.5	2.5	NA	97.6	2.4	NA		95.9	4.1	93.9	6.1	NA	-3.3		-0.9	3.6
Madhya Pradesh	94	6	89.7	10.3	4.3	94.3	5.7	93.7	6.3	89.8	10.2	89.7	10.3	4.5	4	94.5	5.5	95	88.4	11.6	86.9	13.1	92.2	7.8	89.7	83.7	16.3	79.3	20.7	2.3	4.7	7.6	94.6	5.4	93.9	6.1	93.9	6.1	88.2	11.8	0.7	5.7	93.1	6.9	94.1	95.4	94.5	93.7	93.3	6.7	82.4	17.6	91.3	91.8	96	95.7	4.3	10.7	-2.4	93.9	6.1	95.9	4.1	NA	NA	97.9	NA	89.6	10.4	90.7	9.3	NA	NA	NA	NA	4.3	5.2	94.6	5.4	91.8	8.2	94.9	5.1	94.4	5.6	93.3	89.4	10.6	84.2	15.8	91.8	8.2	93	7	78.7	5.2	7.6	3.1	1.4
Maharashtra	92.4	7.6	85.8	14.2	6.6	94.8	5.2	89.9	10.1	86.4	13.6	85.2	14.8	8.4	4.7	95.2	4.8	89.9	91.2	8.8	NA		85.8	14.2	86.2	84	16	NA		9.4	7.2		89.8	10.2	94.3	5.7	84.5	15.5	86.8	13.2	5.3	7.5	90.5	9.5	92.4	86.9	95.6	93.9	92.3	7.7	80.1	19.9	87.7	86	88.9	85	15	10.4	7.3	94	6	83.7	16.3	NA	92	NA	NA	88.1	11.9	77.9	22.1	NA	83.3	NA	NA	5.9	5.8	89.6	10.4	95.3	4.7	94.5	5.5	91	9	NA	86.1	13.9	83.2	16.8	86.7	13.3	86.4	13.6	NA	3.5	12.1	7.8	4.6
Manipur	93.4	6.6	90.4	9.6	3	94.6	5.4	92.2	7.8	91.3	8.7	89.5	10.5	3.3	2.7	95.9	4.1	96.8	77	23	NA		95.8	4.2	90.9	77.3	22.7	73.2	26.8	0.1	-0.3		96.4	3.6	92	8	95.1	4.9	88.1	11.9	1.3	3.9	92.5	7.5	84	93	92.9	94.3	96.3	3.7	69.7	30.3	76.5	89.3	99	97.1	2.9	22.8	-0.8	97.2	2.8	89.4	10.6	87.8	NA	98.9	NA	97.9	2.1	80.6	19.4	84	NA	97.7	NA	-0.7	8.8	93.6	6.4	87.9	12.1	92.8	7.2	97.7	2.3	NA	93.7	6.3	82.9	17.1	94.7	5.3	95.5	4.5	NA	-0.1	5	-1.9	2.2
Meghalaya	82.2	17.8	83.1	16.9	-0.9	82.1	17.9	82.3	17.7	80.6	19.4	85.5	14.5	1.5	-3.2	87.3	12.7	81.5	83.8	16.2	72.5	27.5	87.3	12.7	82.4	79.9	20.1	80.7	19.3	0	3.9	-8.2	77.2	22.8	82.9	17.1	93.9	6.1	81.4	18.6	-16.7	1.5	76	24	83.7	81.7	82.7	90.8	81.9	18.1	70.8	29.2	83.7	83.3	81.7	97.3	2.7	5.2	-15.4	94.2	5.8	NA		81.6	NA	79.6	NA	94.8	5.2	94.9	5.1	81.1	NA	85.4	NA	-0.6		NA		82	18	NA		83.6	16.4	NA	NA		82	18	NA		95.1	4.9	NA		0		-11.5
Mizoram	85.5	14.5	75.5	24.5	10	86.9	13.1	84.1	15.9	70.2	29.8	80.7	19.3	16.7	3.4	86.3	13.7	86.9	80.1	19.9	NA		80.7	19.3	75	69.8	30.2	66.9	33.1	5.6	10.3		83.9	16.1	86.9	13.1	79.2	20.8	71.7	28.3	4.7	15.2	74.2	25.8	77.6	83.5	96.6	80.2	85.8	14.2	44.2	55.8	74.7	74.6	82.6	83.4	16.6	30	2.4	NA		NA		86	77.1	NA	NA	NA		NA		76.4	49.8	NA	NA			NA		NA		NA		NA		NA	NA		NA		NA		NA		NA				
Nagaland	84.2	15.8	68.1	31.9	16.1	86.9	13.1	81.5	18.5	67.6	32.4	68.8	31.2	19.3	12.7	87	13	86.5	72.4	27.6	80	20	78.5	21.5	66.3	61.9	38.1	52	48	8.5	10.5	28	90.7	9.3	81.8	18.2	75.7	24.3	65.3	34.7	15	16.5	88.1	11.9	81.8	81.2	79.7	83.9	90.6	9.4	46.2	53.8	64.3	70.5	70.7	90.1	9.9	41.9	0.5	NA		NA		NA	NA	NA	NA	62.2	37.8	37.7	62.3	70	NA	NA	NA			NA		NA		NA		NA		NA	38.5	61.5	70.5	29.5	NA		NA		NA				
NCT of Delhi	93.7	6.3	92.7	7.3	1	94.6	5.4	92.7	7.3	NA		NA				93.8	6.2	94.8	87.7	12.3	NA		NA		NA	NA		NA					93.4	6.6	100	0	NA		NA				93.4	6.6	NA	94.5	92.9	94.7	93.3	6.7	NA		NA	NA	NA	NA				93.5	6.5	94.2	5.8	NA	NA	NA	NA	NA		NA		NA	NA	NA	NA			95.1	4.9	NA		96.1	3.9	91.8	8.2	93.3	NA		NA		NA		NA		NA				
Odisha	97.3	2.7	93.1	6.9	4.2	97.5	2.5	96.9	3.1	94.1	5.9	92	8	3.4	4.9	97.9	2.1	96.9	96.2	3.8	NA		94.1	5.9	92.4	93.1	6.9	90.2	9.8	3.8	3.1		98.6	1.4	97	3	92.3	7.7	93.3	6.7	6.3	3.7	95.7	4.3	98.4	96.5	97.8	97.8	98	2	89.9	10.1	88.4	95.1	96.1	93.4	6.6	5.8	4.6	97.3	2.7	NA		98.5	NA	NA	NA	93.4	6.6	87.2	12.8	89.6	NA	NA	NA	3.9		98.7	1.3	96.6	3.4	97.1	2.9	96.8	3.2	NA	94.1	5.9	90.9	9.1	96.6	3.4	99.7	0.3	NA	4.6	5.7	0.5	-2.9
Punjab	93.7	6.3	96.7	3.3	-3	93.8	6.2	93.6	6.4	97.2	2.8	96	4	-3.4	-2.4	95	5	94.5	78.3	21.7	NA		98.4	1.6	97.2	86.9	13.1	NA		-3.4	-8.6		92.3	7.7	94.5	5.5	95.3	4.7	97.6	2.4	-3	-3.1	91.5	8.5	NA	96.6	97.1	95.7	91	9	88.9	11.1	NA	97.2	99.1	98.8	1.2	2.6	-7.8	92.4	7.6	NA		NA	NA	NA	95.2	95.6	4.4	83.9	16.1	NA	NA	NA	98	-3.2		93.1	6.9	NA		96.1	3.9	95.1	4.9	NA	96.6	3.4	NA		91.7	8.3	99.7	0.3	NA	-3.5		4.4	-4.6
Rajasthan	94.6	5.4	86.1	13.9	8.5	94.5	5.5	94.7	5.3	85.1	14.9	87.1	12.9	9.4	7.6	94.8	5.2	94.8	93.9	6.1	91.1	8.9	88.6	11.4	87.6	79.1	20.9	64.6	35.4	6.2	14.8	26.5	95.5	4.5	94.4	5.6	93	7	84.1	15.9	2.5	10.3	93.2	6.8	93.1	94.2	97	94.7	95.2	4.8	77.7	22.3	88.6	89.6	91.4	96.9	3.1	15.5	-1.7	94.9	5.1	90.8	9.2	NA	NA	NA	100	87.4	12.6	75.5	24.5	NA	NA	NA	97.4	7.5	15.3	95.1	4.9	94.1	5.9	94.5	5.5	95.2	4.8	NA	87.3	12.7	81.5	18.5	86.3	13.7	88.8	11.2	81	7.8	12.6	8.2	6.4
Sikkim	95.1	4.9	98.9	1.1	-3.8	96.3	3.7	94.2	5.8	98	2	100	0	-1.7	-5.8	95.5	4.5	94.1	NA		NA		100	0	97.4	NA		NA		-4.5			NA		NA		98.2	1.8	99.2	0.8			NA		90	NA	95.3	NA	100	0	NA		NA	100	NA	97.4	2.6		2.6	94.8	5.2	NA		NA	100	NA	NA	100	0	NA		100	98.3	NA	NA	-5.2		NA		94.2	5.8	93.3	6.7	NA		NA	100	0	NA		98.2	1.8	98.1	1.9	NA			-4.9	
Tamil Nadu	97.5	2.5	92.4	7.6	5.1	97.1	2.9	97.9	2.1	93.7	6.3	91	9	3.4	6.9	96.8	3.2	98	NA		NA		92.1	7.9	92.6	91	9	NA		4.7			96.7	3.3	98.1	1.9	93.3	6.7	91.6	8.4	3.4	6.5	91.7	8.3	100	96.8	99.4	95.8	97.6	2.4	92.1	7.9	94.4	92.5	89.7	93.8	6.2	-0.4	3.8	97.2	2.8	100	0	100	NA	NA	NA	92.2	7.8	91.8	8.2	95.5	NA	NA	NA	5	8.2	98	2	NA		97.2	2.8	100	0	NA	91.8	8.2	83.1	16.9	93	7	88.5	11.5	NA	6.2		4.2	11.5
Telangana	92.5	7.5	95.6	4.4	-3.1	93.2	6.8	91.7	8.3	96.2	3.8	94.9	5.1	-3	-3.2	92.4	7.6	92.5	93.1	6.9	NA		95.5	4.5	95.5	NA		NA		-3.1			88.9	11.1	94.5	5.5	94.6	5.4	96.6	3.4	-5.7	-2.1	94.3	5.7	92.7	94.6	85.4	94.1	91.5	8.5	96.2	3.8	NA	96	96.1	94.1	5.9	-1.9	-2.6	93.6	6.4	85.5	14.5	89.8	NA	NA	NA	95.4	4.6	96.1	3.9	NA	NA	NA	NA	-1.8	-10.6	94.2	5.8	91.4	8.6	94.6	5.4	73.2	26.8	NA	96.1	3.9	100	0	94.1	5.9	98.6	1.4	NA	-1.9	-8.6	0.5	-25.4
Tripura	94.6	5.4	80.4	19.6	14.2	94.4	5.6	94.7	5.3	83.3	16.7	77.8	22.2	11.1	16.9	95.4	4.6	95.1	NA		NA		81.6	18.4	81.9	NA		NA		13.8			95	5	94.4	5.6	88.9	11.1	77.5	22.5	6.1	16.9	92.9	7.1	93.8	94.9	94.1	96	96.4	3.6	36.1	63.9	76.9	85.4	79.8	91.9	8.1	56.8	4.5	94.7	5.3	93.5	6.5	NA	NA	NA	NA	79.5	20.5	88.9	11.1	NA	NA	NA	NA	15.2	4.6	98	2	91.4	8.6	96.4	3.6	93.6	6.4	NA	84.5	15.5	66.7	33.3	83.6	16.4	90.1	9.9	NA	13.5	24.7	12.8	3.5
Uttar Pradesh	90.9	9.1	83.7	16.3	7.2	90.6	9.4	91.1	8.9	85	15	82.4	17.6	5.6	8.7	92.3	7.7	90.7	88.9	11.1	87.3	12.7	88.8	11.2	84.6	78.4	21.6	68.1	31.9	3.5	10.5	19.2	88.1	11.9	91.6	8.4	84	16	83.7	16.3	4.1	7.9	89.9	10.1	89	89.6	92.5	92.6	91.1	8.9	75	25	81.9	87.2	90.7	94.2	5.8	14.9	-3.1	91.8	8.2	86.2	13.8	NA	NA	NA	NA	86.3	13.7	74	26	NA	NA	NA	NA	5.5	12.2	91.1	8.9	84.3	15.7	90.9	9.1	91.2	8.8	NA	86	14	69.7	30.3	83.1	16.9	84.2	15.8	NA	5.1	14.6	7.8	7
Uttarakhand	94.8	5.2	90.9	9.1	3.9	95.5	4.5	94.1	5.9	90.3	9.7	91.5	8.5	5.2	2.6	95.2	4.8	95.2	94.5	5.5	*		93.2	6.8	90.5	86.2	13.8	85.5	14.5	2	8.3		93.8	6.2	95.2	4.8	91.8	8.2	90.5	9.5	2	4.7	87.7	12.3	*	94.4	94	97.8	96.4	3.6	86.4	13.6	84.1	88.3	94.6	96.3	3.7	1.3	0.1	95.1	4.9	93.1	6.9	NA	NA	NA	*	92.3	7.7	85.8	14.2	NA	NA	NA	NA	2.8	7.3	92.9	7.1	NA		94.5	5.5	96.1	3.9	NA	87.8	12.2	94	6	89.4	10.6	94.3	5.7	NA	5.1		5.1	1.8
West Bengal	97.7	2.3	96.6	3.4	1.1	98.1	1.9	97.2	2.8	96.6	3.4	96.6	3.4	1.5	0.6	98.6	1.4	96.8	96.2	3.8	NA		97.5	2.5	96	94.8	5.2	NA		1.1	1.4		95.9	4.1	98.3	1.7	93.1	6.9	98	2	2.8	0.3	94.8	5.2	100	96.8	99.6	98.6	95.7	4.3	96.1	3.9	99.4	96.6	95.2	95.8	4.2	-1.3	-0.1	97.3	2.7	98.2	1.8	NA	NA	NA	NA	96.8	3.2	95.8	4.2	NA	NA	100	NA	0.5	2.4	98.4	1.6	97.7	2.3	100	0	96.7	3.3	NA	95.8	4.2	96.6	3.4	95.8	4.2	97	3	100	2.6	1.1	4.2	-0.3

Gender and Zero-Dose Status

Of the 29 states for which data are available in NFHS 5 and NFHS 4, eight (28%) states reported a female ZD prevalence of 0 to <5%, 16 (55%) states a prevalence of 5% to <10%, two (7%) states, and three (10%) states a prevalence of 10 to <15% and 15% and above, and in NFHS 5, the corresponding figures for NFHS 4 being 28%, 28%, 24%, and 21%, respectively, for the four categories. The maximum reduction among female ZD proportions has been achieved in Arunachal Pradesh (19.2%), Tripura (16.9%), Nagaland (12.7%), Assam (11.6%), and Uttar Pradesh (8.7%). Eight states reported an increase in prevalence between the two rounds. These are Andhra Pradesh, Chhattisgarh, Jharkhand, Punjab, Sikkim, Telangana, Kerala, and Meghalaya.

Male ZD prevalence of 0 to <5% was reported across nine (31%) states, 5 to <10% in 16 (55%) states, 10 to <15% in three (10%) states, and a prevalence of 15% and above in one (3%) states of the country. Overall, 28% of states reported a prevalence of 0 to <5% in NFHS 4, with 24% each being reported for the other three categories. The maximum improvement in male ZD prevalence was reported in Nagaland (19.3%), Arunachal Pradesh (17.4%), Mizoram (16.7%), Tripura (11.1%), and Rajasthan (9.4%). Six states reported an increase in prevalence between the two rounds. These are Andhra Pradesh, Chhattisgarh, Jharkhand, Punjab, Sikkim, and Telangana.

Overall female ZD prevalence was less than male ZD across 10 (34%) states of the country as per NFHS 5, a dip of 4% compared to NFHS 4. The disparity between the two rounds was reduced more for male ZD compared to female ZD prevalence. While the prevalence for male ZD ranged from 32.4% (Nagaland) to 1.5% (Chhattisgarh) in NFHS 4, the same reduced to 17.9% (Meghalaya) to 0.7% (Himachal Pradesh) in NFHS 5. Similarly, the prevalence of female ZD ranged from 32.8% (Arunachal Pradesh) to 2.6% (Chhattisgarh) in NFHS 4 and from 18.5% (Nagaland) to 2.1% (Tamil Nadu) in NFHS 5. The maximum disparity between male and female ZD prevalence was reported by Nagaland (5.4%), Maharashtra (4.9%), and Bihar (3.4%) in NFHS 5.

Mother’s Education and Zero-Dose Status

The article provides a comparative analysis for ZD children wherein mothers who have had no schooling with respect to mothers who had 12 or more years of schooling. For mothers with no schooling, data were analyzed for 26 states for NFHS 5 and 25 states for NFHS 4. Data were analyzed for 29 and 28 states, respectively, for mothers with 12 or more years of schooling for NFHS 5 and NFHS 4, respectively.

For mothers with no schooling, three (12%) states reported a prevalence of 0 to <5%, 15 (58%) a prevalence of 5 to <10%, five (19%) a prevalence of 10 to <15%, and three (12%) states a prevalence of 15% or more in NFHS-5, and the corresponding figures in NFHS 4 were three (12%), three (12%), six (24%), and 13 (52%) for the four cohorts. Twelve (41%) states reported a ZD prevalence of 0 to <5%, 15 (52%) a prevalence of 5% to <10%, and one (3%) state each a prevalence of 10% to <15% and 15% and above for mothers with 12 or more years of schooling in NFHS 5. Similarly, prevalence across the four cohorts in NFHS 4 was 14 (50%) states, 10 (36%) states, and two (7%) states for mothers with 12 or more years of schooling.

ZD prevalence in mothers with no schooling has shown a substantial decline between the two rounds with Tripura (56.8%), Nagaland (41.9%), Mizoram (30%), Arunachal Pradesh (27.7%), and Manipur (22.8%) reporting the maximum improvement. In comparison, improvement in the other group was on the lower side. The maximum reductions were reported in Maharashtra (7.3%), Assam (5.2%), Odisha (4.6%), Tripura (4.5%), and Karnataka (4.4%). While the prevalence of ZD children in families where mothers have not received any schooling remains high, the disparities have reduced significantly between NFHS 4 and 5. While the range was 63.9% (Tripura) to 3.8% (Tamil Nadu) in NFHS 4, it has been reduced to between 25.8% (Mizoram) and 3.9% (Jammu & Kashmir and Karnataka) in NFHS 5. On the other hand, the disparities have increased in families with mothers having 12 or more years of schooling. This is reflected through a range of 18.1% (Meghalaya) to 2.0% (Odisha) in NFHS 5 compared to 16.6% (Mizoram) and 0.9% (Andhra Pradesh) in NFHS 4. In addition, five states have reported an increase in ZD prevalence in mothers with no schooling group, and 14 states reported an increase in mothers with 12 or more years of schooling group.

Birth Order and Zero-Dose Status

The data for this parameter are fragmented across the two surveys. While data for birth order one has been analyzed for 29 states for NFHS 4 and 5, the analysis for birth order four to five is based on 23 states, and that for birth order six or more for 17 states in NFHS 4 and 11 states in NFHS 5.

In NFHS 5, 13 (45%) states have reported a ZD prevalence of 0 to <5% for birth order one, 12 (41%) a prevalence of 5% to <10%, and 4 (14%) states a prevalence of 10% to <15%. The corresponding figures for NFHS 4 were 11 (38%), eight (28%), and six (21%). In addition, four (14%) states in NFHS 4 had reported a prevalence of 15% or higher. For birth order four to five in NFHS 5, two (9%) states reported a prevalence of 0 to <5%, 11 (48%) a prevalence of 5% to <10%, and four (17%) and six (26%) states a prevalence of 10% to <15% and 15% and higher, respectively. The figures in NFHS 4 for the four cohorts were one (4%), five (22%), five (22%), and 12 (52%). Similarly, for birth order six and above, a ZD prevalence of 5% to <10% was reported by one (9%) state in NFHS 5 and two (12%) states in NFHS 4. Three (27%) and two (12%) states reported a prevalence of 10% to <15%, and seven (64%) and 13 (76%) states, a prevalence of 15% and above.

Disparities have significantly reduced for all the above three birth orders between the two rounds, with maximum reductions being observed for higher birth orders. This is evident from the reduction in the range observed for these cohorts, while for birth order six or more the highest prevalence was 59.6% in Arunachal Pradesh and the lowest 6.9% in Chhattisgarh (NFHS 4), the highest prevalence has reduced to 28.3% in Assam in NFHS-5. The lowest prevalence reported in NFHS-5 though has seen a rise with it being lowest in Rajasthan (8.9%). The range for birth order four to five has similarly reduced from 44.4% (Arunachal Pradesh) to 1.6% (Chhattisgarh) in NFHS 4 to 27.6% (Nagaland) to 3.8% (Odisha) in NFHS 5. The corresponding figures for birth order one are 21.50% (Nagaland) to 0.50% (Chhattisgarh) and 13.70% (Mizoram) to 1.40% (West Bengal) for the two survey periods. The maximum reduction in ZD prevalence for birth order six and more is observed in Arunachal Pradesh (35.6%), Nagaland (28.0%), Rajasthan (26.5%), Uttar Pradesh (19.2%), and Haryana (10.2%) between NFHS 4 and NFHS 5 and in Arunachal Pradesh (29.6%), Rajasthan (14.8%), Assam (13.2%), Haryana (10.7%), and Nagaland and Uttar Pradesh each (10.5%) for birth order four to five. Tripura (13.8%), Maharashtra (9.4%), Nagaland (8.5%), Arunachal Pradesh (7.6%), and Rajasthan (6.2%) reported the maximum reduction between the two rounds for birth order one.

The ZD prevalence has increased for birth order one in seven states, for four to five in three states, and for six and above across three states. The prevalence for all three cohorts has increased in Chhattisgarh. In addition, Manipur reports the maximum gap in ZD prevalence between birth order four to five (23.0%) and birth order one (4.1%), and Assam the maximum difference between birth order six and above (28.3%) and birth order one (8.6%) in NFHS-5.

Religion and Zero-Dose Status

Data for religion as an equity determinant is available for Buddhists, Christians, Hindus, Muslims, Sikhs, and others in NFHS rounds. However, due to the unavailability of data for multiple states, the current analysis has been undertaken for only Hindu and Muslim children. Data for Hindu children are available for 26 states under NFHS 5 and for 27 states under NFHS 4, and for Muslim children, it is available for 20 and 24 states, respectively.

Nine (35%) states in the country reported a ZD prevalence of 0 to <5%, 16 (62%) a prevalence of 5% to <10%, and one (3%) state a prevalence of 10% to <15% for Hindu children in NFHS 5. The corresponding figures for NFHS 4 are nine (33%) each for the 0 to <5% and 5% to <10%, six (22%) a prevalence of 10% to <15%, and three (11%) states a prevalence of 15% and above. Five (25%) states reported a ZD prevalence of 0 to <5% in NFHS 5 for Muslim children, seven (35%) a prevalence of 5% to <10%, six (30%) a prevalence of 10% to <15%, and two (10%) states a prevalence of 15% and above. The figures for NFHS 4 for Muslim children were four (17%), seven (29%), three (13%), and 10 (42%), respectively.

Tripura (15.2%), Arunachal Pradesh (9.7%), Rajasthan (7.5%), Gujarat (7.2%), and Karnataka and Maharashtra (both 5.9% each) were the states which have reported the maximum reduction in the proportion of ZD among Hindu children, while Haryana (18.4%), Assam (15.8%), Rajasthan (15.3%), Uttar Pradesh (12.2%), and Manipur (8.8%) reported the highest declines among Muslim children. Overall, 40% of the states in the country reported a lower ZD prevalence among Muslim children compared to Hindu children up from 29% in NFHS 4. While the prevalence of ZD among Hindu children increased across seven states between the two NFHS rounds, a rise among Muslim children was reported from three states of the country. The interstate disparity has reduced significantly among Muslim children, while the range was 62.3% (Nagaland) to 3.9% (Tamil Nadu) in NFHS 4, it has reduced to 16.3% (Maharashtra) to 1.8% (Tamil Nadu) in NFHS 5. The figures for Hindu children across the two rounds are 37.8% (Nagaland) and 1.9% (Chhattisgarh) in NFHS 4 and 11.1% (Arunachal Pradesh) and 2.1% (Himachal Pradesh).

Caste and Zero-Dose Status

Data for social caste and ZD status has been analyzed for 25 states for OBC across both the NFHS rounds, for 25 states in NFHS-5 and 26 states in NFHS-4 for SC, and 22 and 23 states, respectively for ST in NFHS 5 and NFHS 4 and 26 states across both the rounds for others.

Among OBCs, 10 (40%) and 15 (60%) states reported a ZD prevalence of 0 to <5% and 5% to <10% in NFHS 5, while eight (32%) states each reported similar prevalence in NFHS 4. Six (24%) and three (12%) states reported a prevalence of 10% to <15% and 15% and above in NFHS 4. Ten (40%), 13 (52%), and one (4%) state each reported a prevalence of 0 to <5%, 5% to <10%, 10% to <15%, and 15% and above among SC in NFHS 5, respectively, the corresponding figures in NFHS 4 being seven (27%), seven (27%), nine (35%) and three (12%) for the four cohorts. In the ST category, four (18%) states reported a prevalence of 0 to <5%, 14 (64%) states a prevalence of 5% to <10%, and two (9%) states each a prevalence of 10% to <15% and 15% and above in NFHS 5. The corresponding figures in NFHS 4 were four (17%), five (22%), three (13%), and 11 (49%) for the four cohorts, respectively. In the others category in NFHS 5, 10 (38%) states, 12 (46%) states, and two (8%) states each reported prevalence for the four cohorts with the corresponding figures being nine (35%), seven (27%), eight (31%), and two (8%) in NFHS 4.

As with other equity determinants, the disparities for the OBC, SC, and ST groups have reduced between the two NFHS rounds. While for OBCs the range of disparity has reduced from 9.1% (Uttar Pradesh) to 2.8% (Tamil Nadu) and from 16.9% (Uttar Pradesh) to 1.4% (Jammu & Kashmir) between NFHS 4 and NFHS 5, this reduction for SC is from 61.50% (Nagaland) to 0.30% (Chhattisgarh) in NFHS 4 to 17.10% (Arunachal Pradesh) to 1.30% (Odisha) in NFHS 5. Among the ST groups, the range has reduced from 38.6% (Arunachal Pradesh) to 0.3% (Himachal Pradesh) in NFHS 4 to 18.0% (Meghalaya) to 0.2% (Himachal Pradesh) in NFHS 5. The disparity for the other caste-based category has increased between the two rounds from 23.8% (Assam) to 0.3% (Punjab) to 26.8% (Tamil Nadu) to 1.6% (Himachal Pradesh).

Residence and Zero-Dose Status

Data for this equity determinant are available for 28 states for NFHS 5 and 29 states for NFHS 4 datasets. In the urban geographies of the country, seven (25%) states reported a ZD prevalence of 0 to <5%, 14 (50%) a prevalence of 5% to <10%, five (18%) a prevalence of 10% to <15%, and two (7%) states a prevalence of 15% and above in NFHS 5. The corresponding figures for NFHS 4 were eight (28%), 12 (41%), four (14%), and five (17%), respectively. In the rural geographies of the country, NFHS 5 reported a ZD prevalence of 0 to <5% across 10 (36%) states, while 14 (50%) states reported a prevalence of 5% to <10%, and two (7%) states each a prevalence of 10% to <15% and 15% and above. The prevalence reported in NFHS 4 was eight (28%), seven (24%), five (17%), and nine (31%) for the four categories starting from 0 to <5%.

Arunachal Pradesh and Nagaland reported the maximum improvement in ZD prevalence across both urban and rural geographies. While the urban and rural ZD prevalence has reduced by 15.5% and 19.9% in Arunachal Pradesh, respectively, the reduction in Nagaland was 15.0% and 16.5%, respectively. Other states which reported appreciable declines in urban ZD proportions were Haryana (8.9%), Uttar Pradesh (7.8%), and Himachal Pradesh (7.2%). Rural ZD proportion declined significantly in the northeastern states of Tripura (16.9%), Mizoram (15.2%), and Assam (10.7%). Overall, 34% of states in NFHS 5 reported a lower urban ZD prevalence compared to rural ZD, a decline of 32% compared to NFHS 4. Urban ZD prevalence increased in nine states compared to four states which reported a rise in rural ZD prevalence between NFHS 4 and 5.

The interstate disparities reflect a more pronounced decline for rural ZD compared to urban ZD between the two rounds. While the highest prevalence of rural ZD reduced from 34.7% (Nagaland) to 18.2% (Nagaland), the same reduced from 24.3% (Nagaland) to 22.8% (Meghalaya) for urban ZD proportions.

The states of Andhra Pradesh, Chhattisgarh, and Punjab reported an increase in the prevalence of ZD children in both urban and rural geographies over the two rounds. In the urban pockets, ZD proportions increased from 2.3% to 10.6% in Andhra Pradesh, 1.5% to 8.2% in Chhattisgarh, and 4.7% to 10.2% in Punjab. The corresponding increase in rural geographies was 3.9% to 5.4%, 2.2% to 3.9%, and 2.4% to 6.6% for the three states, respectively. Jharkhand is another state which has reported an increase in urban ZD proportions (3.4% to 12.8%) with the progress across rural geographies stagnating (6.8% to 6.5%) between the two rounds. Similarly, the proportion of ZD in Meghalaya increased by 16.7% in NFHS 5 compared to NFHS 4.

Zero-Dose and Key Programmatic Interventions

Table 3 represents the correlation coefficient between ZD and key programmatic components. It is evident that a lower proportion of first-trimester registration, four or more ANC visits, and institutional deliveries is associated with higher chances of children being ZD, with the strength of this negative association increasing between the two NFHS rounds (Table 3).

**Table 3:** Association between ZD and key MCH indicators ZD = zero dose; NFHS = National Family Health Survey; ANC = antenatal care; MCH = maternal and child health

Indicator	Zero dose	
	NFHS 4	NFHS 5
Four or more ANC visits	r = -0.56; p = 0.0017	r = -0.57; p = 0.0009
First-trimester registration	r = -0.42; p = 0.0216	r = -0.56; p = 0.0013
Institutional deliveries	r = -0.62; p = 0.0003	r = -0.74; p = 0.000002
Stunting	r = 0.05; p = 0.7915	r = 0.31; p = 0.0998
Wasting	r = -0.22; p = 0.2406	r = -0.22; p = 0.2336

## Discussion

As evident, there has been a reduction in ZD prevalence across the country and at state levels between the two NFHS rounds. While the overall rate of reduction is 4.1% between the two rounds, the number of states which reported a lower ZD prevalence of 5% to <10% has increased from nine to 17 and those reporting higher ZD prevalence of 10% and above reduced from 12 to four. The states that report a ZD prevalence of 0 to <5% remain constant with nine states each in NFHS 5 and NFHS 4. Similarly, there has been consistent progress across all the key equity parameters. Moreover, among these parameters, the maximum reduction has been for cohorts that have been traditionally vulnerable and marginalized. For instance, the rate of reduction is 4.9% in rural geographies, 6.7% among Muslim children, 6.5% among ST population groups, 9.4% among children with birth order six and above, 7.0% among mothers with no schooling, and 7.4% among populations in the lowest wealth quintile.

This is reflective of the improved reach of the immunization program in India as evident through recent efforts. These include the Mission Indradhanush (MI) and Intensified Mission Indradhanush (IMI) rounds contributing to a sustained increase in coverage across underperforming districts of the country as is evident from an 18.5% increase in full immunization coverage (FIC) across 190 districts which conducted IMI rounds in 2017-2018 [11]. Similarly, investments in optimizing last-mile delivery of vaccines to session sites with the electronic Vaccine Intelligence Network (eVIN) [12,13] and the introduction of multiple new vaccines have further strengthened immunization systems across the country [14].

However, what the data also reflect are the challenges and disparities that still surmount the immunization program in India. Five states of the country with high Pentavalent 1 coverage rates have reported an increase in ZD prevalence. This ranges from 0.5% in Odisha to 3.5% in Andhra Pradesh. Similarly, while states across the country have had a decline in ZD prevalence in line with earlier NFHS rounds, pockets in the country continue to report high proportions. Bihar, Madhya Pradesh, and Uttar Pradesh, which account for close to 40% of the national infant cohort in India, each report a prevalence of 6% or higher, with Uttar Pradesh reporting a high prevalence of 9.1%. Four of the northeastern states (Arunachal Pradesh, Meghalaya, Mizoram, and Nagaland) continue to report a prevalence of more than 10%, with Assam, the largest northeastern state, reporting a prevalence of 9.3%. In line with earlier findings, the urbanized states of Andhra Pradesh, Gujarat, Maharashtra, and Telangana have a prevalence of 7.0% or more [15]. Inter-district disparities also need to be studied, with more than 145 (21.3%) districts reporting a prevalence of more than 10%. In addition, certain states such as Andhra Pradesh, Chhattisgarh, Jharkhand Meghalaya, Punjab, and Telangana have slipped across multiple equity parameters. The sub-national approach with a clear focus on geographies with the highest number and prevalence of ZD that the GAVI 5.0 strategy advocates for is therefore a step in the right direction [5].

The equity parameters also reflect a contrasting picture with a slow-footed reduction among ZD children for girl children, across urban geographies, first-born children, mothers with 12 or more years of schooling, and children in families with the highest wealth quintiles. This is expected as some of the abovementioned parameters have had lower baselines; however, the comparatively slow pace of reduction among girl children and urban geographies is particularly concerning.

This article tries to analyze the impact of gender by examining the difference in ZD proportions among male and female children, the impact of maternal education, the birth order of the children, and touchpoints with health services as important predictors. Often gender is not identified as a major determinant for tracking immunization performance as the coverage rates among boys and girls remain almost the same [15,16]. However, the disparity remains evident, as illustrated in the current analysis. While the ZD proportions among male and female children have reduced, the pace has been slow for female children. In addition, only 10 states of the country report a lower prevalence among female children compared to male children as per NFHS 5, a dip of 4% compared to NFHS 4. This difference has important programmatic implications as it points toward possible challenges at the individual, family, household, and community levels that might impede access for female children [17]. With strong outreach immunization programs across many countries, while the coverage for girls improves over time (into their first and second year), the difference in ZD proportions might contribute to continued inequities for health interventions and services wherein delivery systems might not be as mature for immunization.

Maternal education has always been identified as an important positive predictor of immunization coverage with its impact more profound in low and low-middle-income countries (LMICs) [18,19]. While earlier work in India [8] identified children of mothers with no schooling as having a significant association with ZD proportions, of all the equity parameters studied in this article, the maximum improvement in the reduction of ZD prevalence has been noticed among mothers with no schooling. This is reflective of a continued improvement in healthcare service delivery and health literacy through a strong focus on community mobilization and engagement using the Accredited Social Health Activist (ASHA) network in the country. It is also in concordance with earlier evidence which states that, as opposed to formal education, health literacy and health knowledge are modifiable and can be gained informally [20].

However, despite this reduction, the overall prevalence of ZD children among mothers with no schooling remains high. Moreover, 14 states of the country have reported an increase in ZD prevalence among mothers with 12 or more years of schooling, thus implying continued efforts in this direction. Most of the states included in this group are those where overall sociodemographic and public health indicators continue to remain weak, thus pointing toward early trends of changing equity dynamics. In line with other parameters studied, there has been a reduction in ZD prevalence among higher birth orders, thereby further potentiating better outcomes. However, the overall ZD prevalence remains very high for birth orders six, and this is aligned with evidence from other countries [21,22]. This often translates to incomplete immunization schedules, thereby also pointing toward intrafamilial inequities.

Adding to the existing evidence base, the article aims to articulate the impact of access to health services by mothers as an important determinant of coverage [23,24]. A negative correlation is established between first-trimester registration, four or more ANC visits, institutional deliveries, and ZD prevalence. This negative correlation has increased between the two NFHS rounds, possibly pointing toward increasing marginalization of vulnerable population groups as coverage of health services continues to rise.

Of all three indicators, the strongest correlation has been observed for institutional deliveries. A continued push toward improved maternal care services will have a definite impact on reducing ZD proportions. Moreover, efforts should be continued on ensuring that mothers with limited exposure to healthcare services be reached, a visit by the ASHA during the eighth or ninth month of pregnancy can be undertaken to promote institutional deliveries in pockets where ANC coverage continues to be low and home deliveries continue to be high. This has been demonstrated through programs advocating for community-based distribution of misoprostol for home deliveries which simultaneously had a positive impact on institutional deliveries [25]. Similarly, the comprehensive home visitation programs in India (Home-Based Care of the Newborn (HBNC) [26] and Home-Based Care for Young Child (HBYC) [27]) should be leveraged to reduce ZD proportions. Specifically, the HBNC program with a sequence of six (for institutional births) and seven scheduled visits (for home births) by ASHAs to newborns from birth to 42 days of birth is an optimal platform to advocate for immunization services.

Although it is statistically not significant, the correlation between stunting and ZD children has been found to be positive and increasing, thereby articulating the long-term impact of ZD children missing out on other life course entitlements [15]. While there is a negative association with wasting, this appears to be minimal and needs to be studied further.

Religion, caste, and socioeconomic status are the other equity parameters that have often been studied in relation to ZD proportions. While data availability across religions has limited comparisons to Hindu and Muslim children, data for wealth quintiles are only available at the national level. In line with other parameters, there has been improvement across states and population groups that have been traditionally disadvantaged. The improvement among Muslim children and SC and ST population groups has been on the higher side, again reiterating the improved reach of the program. A recent article in BMJ Global Health which studied the impact of ethnicity on ZD prevalence across 62 countries and used caste as a marker for ethnicity for India illustrates that differences among ethnic groups persisted after adjustment for wealth, maternal education, and area of residence [28]. Similarly, Muslim children have been documented to have lower immunization coverage both across India [29,30] and other countries. While there has been a significant decline in ZD prevalence among Muslim children, efforts to further enhance their engagement with the program need to be continued.

Variations in rural and urban ZD proportions are well established in this article. Not only has the overall reduction of ZD proportions in the urban areas been low compared to rural geographies in the country, the overall urban areas now account for a higher proportion of ZD prevalence in India. This mimics the trend for other immunization indicators in the country with the urban FIC now lower than the rural FIC (NFHS 5). In addition, the progress in urban geographies remains slow-footed compared to rural areas (17.9% increment in urban FIC compared to 38.2% in rural FIC between NFHS 3 (2005-2006) [9] and NFHS 5. The urban immunization challenges are in line with other studies which similarly report ZD concentration in urban slums across multiple countries and regions of the world [31,32]. This implies focused efforts to address the urban immunization challenge. The Ministry of Health and Family Welfare, Government of India, has recognized this and recently undertaken efforts to improve immunization coverage across urban areas of the country. This includes a partner consortium-based approach called the City Embrace Model to improve outcomes across 104 high-priority urban cities of the country. The model advocates for not only addressing the supply-side challenges but also builds on a Vaccination on Demand concept to optimize demand. In addition, the engagement of stakeholders beyond the public healthcare delivery system such as urban local bodies, education departments, and the private sector is also prioritized. Simultaneously, a revamp of the National Urban Health Mission is being undertaken, thereby facilitating an overall improvement in the urban primary, secondary, and tertiary care systems.

ZD children and communities, as discussed above, remain functionally invisible to health systems and go without basic vaccines every year. These vaccination blindspots leave millions of children vulnerable to vaccine-preventable diseases. Reaching and integrating ZD children with the health systems remains an unfinished agenda, and it is not about numbers, but individuals, families, and communities who are often left out of the formal healthcare system. Evidence now tells us that focusing on ZD children is particularly important because those who are reached with the first vaccine are highly likely to also receive the remaining vaccines [33].

Highly differentiated strategies are required to successfully reach ZD children and missed communities. Around 50% of ZD children reside in urban settings, remote rural settings, or conflict settings, while the rest of them are not reachable due to factors such as gender, poverty, ethnicity, and other sociocultural barriers. Building on the Identify-Reach-Monitor-Measure-Advocate framework developed by Gavi [34], we propose a 4P approach to improve outcomes, which includes interventions at the Policy level, identifying the Population groups that need to be targeted, leveraging existing Platforms, and establishing Partnerships to accelerate progress. At the Policy level, while the goal remains to reach an FIC of 90%, it is imperative to focus on ZD children and missed communities, with national policies strongly advocating for no child to be left behind. Similarly, incentivizing healthcare workers for identifying ZD children can be looked into. An important aspect will be the financial resources that will be required to pursue this very important agenda, with recent estimates advocating for a rise in resources being allocated to the immunization program in India [35]. The equity indicators provide a fair idea of the population groups and communities that need to be targeted. With immunization coverage increasing over time, identifying and reaching ZD children will require focusing attention on micro clusters and overcoming existing service delivery challenges and vaccine hesitancy in a coordinated manner. Operationally, this will require additional budgets, further stretching of the existing workforce, and population flux issues. Leveraging existing platforms will be needed to address some of the above challenges. Digital approaches which are unitized, unified, ubiquitous, and universal will be required, adapting the CoWIN platform which was established to facilitate the rollout of COVID-19 vaccines in India for routine immunization is already underway [36]. As stated above complementary programs which provide important touchpoints across the continuum of care will need to be leveraged. In addition, new avenues and approaches such as behavioral insights and human-centered design approaches will need to be integrated with program planning and implementation. Surveillance efforts would need to be ramped up to provide timely and granular data to better organize coverage and equity improvement efforts. Above all, partnerships with stakeholders beyond immunization and beyond health will be needed to further expand the reach and ensure a comprehensive sociocultural customized approach to address challenges related to ZD children in the country.

It is also important that the impact of the pandemic is factored in. Globally, it is estimated that the number of ZD children has increased from 13 million in the pre-pandemic period (2019) to 18 million in 2021 [37]. India has also seen a rise with an estimated 2.7 million ZD children in 2021 [7]. This is bound to have an adverse impact on the progress to date and would require renewed efforts to address the backsliding in immunization coverage.

While important insights are gleaned from the analysis, this article has its fair share of limitations. As the analysis has been sourced from NFHS reports and not raw data, the results need to be interpreted with caution. The early trends sourced from NFHS reports need to be complemented with detailed analysis from NFHS raw data to better identify and account for uncertainty estimates and rule out differences and reductions attributable to chance. The use of NFHS reports and the variability in the availability of data for certain equity determinants has also limited analysis to a certain extent, and the NFHS raw data should be further explored to better understand the interplay of the equity determinants at the national, state, and district levels. The high baselines for ZD prevalence across certain states in NFHS 4 also need to be factored in while reflecting on the reduction between the two rounds. Therefore, policy and programmatic decisions need to be accordingly aligned.

## Conclusions

Overall, despite the above limitations, the article provides valuable insights into the current scenario of ZD children in India. The data suggest that while there has been sustained progress at the national, state, and district levels and across key equity determinants for ZD children between 2015-2016 to 2019-2021, critical issues and challenges continue to exist. The pandemic is also bound to have a negative impact on the program. Hence, continued and augmented efforts are needed to further expand the reach of the immunization program. Adopting mechanisms to identify population cohorts beyond the reach of the program, a focus on vaccine demand interventions, and leveraging partnerships with other health programs and stakeholders beyond health is imperative and much needed. This will ensure that ZD population groups are identified and integrated with the health system and the impact of the pandemic is mitigated.
